# The carotenoid biosynthetic and catabolic genes in wheat and their association with yellow pigments

**DOI:** 10.1186/s12864-016-3395-6

**Published:** 2017-01-31

**Authors:** Pasqualina Colasuonno, Maria Luisa Lozito, Ilaria Marcotuli, Domenica Nigro, Angelica Giancaspro, Giacomo Mangini, Pasquale De Vita, Anna Maria Mastrangelo, Nicola Pecchioni, Kelly Houston, Rosanna Simeone, Agata Gadaleta, Antonio Blanco

**Affiliations:** 1 Department of Soil, Plant and Food Sciences, University of Bari ‘Aldo Moro’, Via G. Amendola 165/A, Bari, Italy; 2 Department of Agricultural and Environmental Science, University of Bari ‘Aldo Moro’, Via G. Amendola 165/A, 70126 Bari, Italy; 3 Council for Agricultural Research and Economics - Cereal Research Centre, 71122 Foggia, Italy; 4 The James Hutton Institute, Invergowrie, Dundee, DD2 5DA Scotland

**Keywords:** Wheat, Carotenoids genes, SNP, Association mapping, Yellow pigments, Flour colour

## Abstract

**Background:**

In plants carotenoids play an important role in the photosynthetic process and photo-oxidative protection, and are the substrate for the synthesis of abscisic acid and strigolactones. In addition to their protective role as antioxidants and precursors of vitamin A, in wheat carotenoids are important as they influence the colour (whiteness vs. yellowness) of the grain. Understanding the genetic basis of grain yellow pigments, and identifying associated markers provide the basis for improving wheat quality by molecular breeding.

**Results:**

Twenty-four candidate genes involved in the biosynthesis and catabolism of carotenoid compounds have been identified in wheat by comparative genomics. Single nucleotide polymorphisms (SNPs) found in the coding sequences of 19 candidate genes allowed their chromosomal location and accurate map position on two reference consensus maps to be determined. The genome-wide association study based on genotyping a tetraploid wheat collection with 81,587 gene-associated SNPs validated quantitative trait loci (QTLs) previously detected in biparental populations and discovered new QTLs for grain colour-related traits. Ten carotenoid genes mapped in chromosome regions underlying pigment content QTLs indicating possible functional relationships between candidate genes and the trait.

**Conclusions:**

The availability of linked, candidate gene-based markers can facilitate breeding wheat cultivars with desirable levels of carotenoids. Identifying QTLs linked to carotenoid pigmentation can contribute to understanding genes underlying carotenoid accumulation in the wheat kernels. Together these outputs can be combined to exploit the genetic variability of colour-related traits for the nutritional and commercial improvement of wheat products.

**Electronic supplementary material:**

The online version of this article (doi:10.1186/s12864-016-3395-6) contains supplementary material, which is available to authorized users.

## Background

Carotenoids are organic pigments commonly present in plants, photosynthetic algae and some species of fungi and bacteria. They are normally associated with thylakoid membranes of chloroplasts and often provide the yellow, orange and red pigmentation to many flowers, fruits and roots [1]. In plants, carotenoids play an important role in photosynthesis, photo-oxidative protection [2], and represent the substrate for the synthesis of apocarotenoid hormones, such as abscisic acid and strigolactones [3, 4]. Carotenoid actions and their relation to human health and disease have been widely reviewed [5]. Carotenoids and some of their metabolites are suggested to play a protective role in a number of reactive oxygen species (ROS)-mediated conditions, such as, i.e., cardiovascular diseases, several types of cancer or neurological, as well as photosensitive or eye-related disorders.

**Fig. 1:**
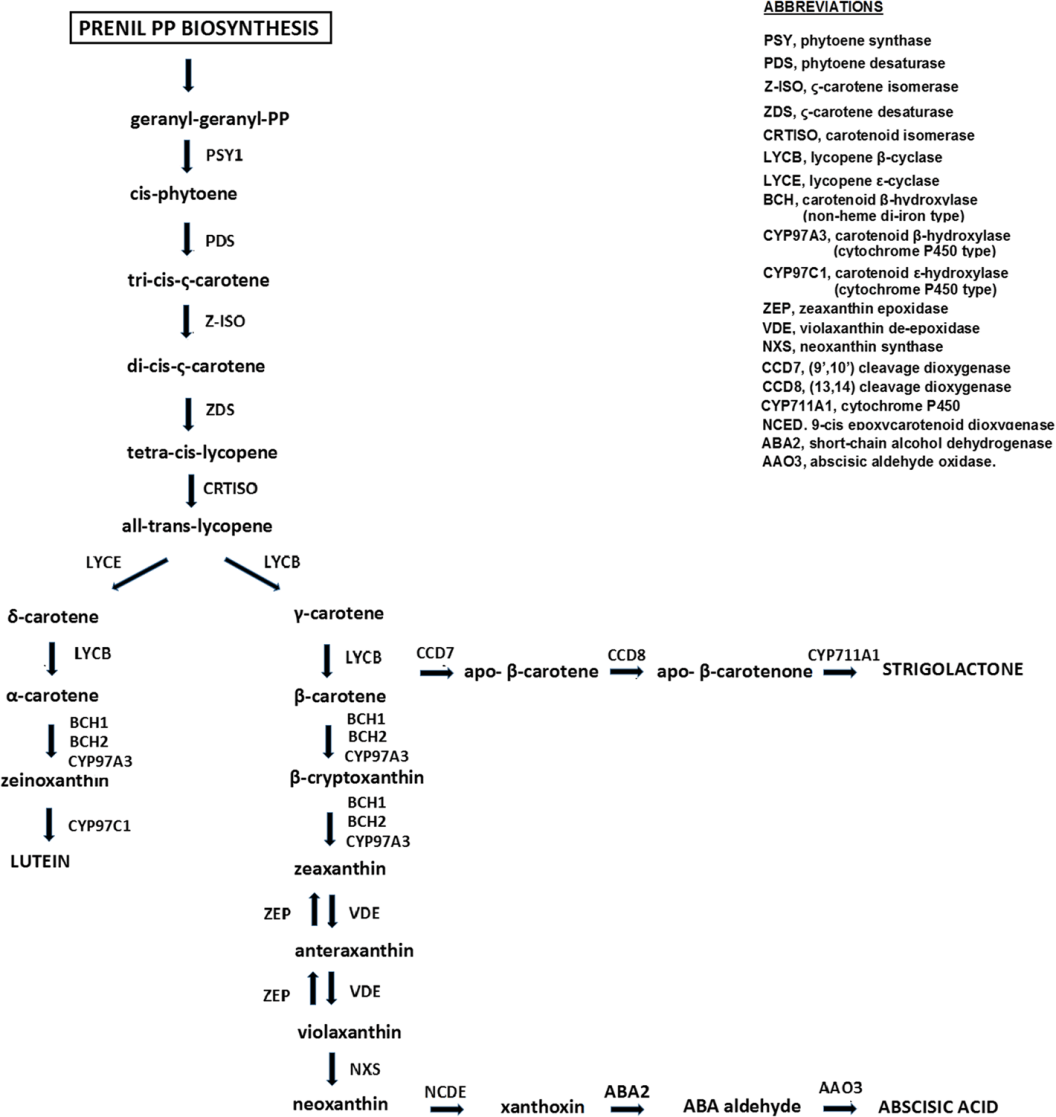
The carotenoid metabolic/catabolic pathway (modified from Vranova [57])

Carotenoids are typically divided into two classes: carotenes, which are tetraterpenoid hydrocarbons, and xanthophylls that contain one or more oxygen groups [6]. The carotenoid biosynthesis has been almost completely elucidated due to work in *Arabidopsis thaliana,* rice, maize and in some ornamental plants [6, 7]. Briefly, the first stage of the biosynthetic process, mediated by phytoene synthase (PSY), involves the condensation of two molecules of geranylgeranyl diphosphate to produce phytoene, which normally does not accumulate in tissues (Fig. 1). In higher plants, the phytoene undergoes a series of four desaturation reactions, mediated by phytoene desaturase (PDS), zeta-carotene isomerase (Z-ISO), zeta-carotene desaturase (ZDS) and carotenoid isomerase (CRTISO) that lead to the production of lycopene. Double lycopene cyclization can produce α-carotene (branch β-ε) or β-carotene (branch β-β). Subsequent modifications transform α-carotene to zeinoxanthin and lutein, and the β-carotene to β-cryptoxanthin, zeaxanthin, antheraxanthin, violaxanthin and neoxanthin. The oxidative cleavage of violaxanthin and neoxanthin form xanthoxin, which is converted to the phytohormone abscisic acid via ABA-aldehide [3]. Strigolactones derive from β-carotenoids via a pathway involving the carotenoid cleavage dioxygenases CCD7, CCD8 and CYP711A1 [4].

Wheat is one of the most important crops worldwide and is the leading source of plant protein in human food, having a higher protein content than other major cereals, such as maize or rice [8]. In addition to their protective role as antioxidant and as precursors of vitamin A, carotenoids are commercially important as they confer whiteness vs. yellowness degree to the end products of wheat. Consumers usually prefer white bread made from common wheat (*Triticum aestivum* L. subsp. *aestivum*), while yellow semolina and pasta made from durum wheat (*Triticum turgidum* L. subsp. *durum*) are preferred by the market. Flour and semolina colour is mainly the result of carotenoid accumulation in the grain [9], but the final colour of end-finished products is also associated to losses during grain storage and to the carotenoid oxidative degradation by enzymes, such as polyphenol oxidase, lipoxygenase and peroxidase, during processing [10, 11].

Flour and semolina colour in wheat is a quantitative trait controlled by several genes with additive effect, and influenced by environmental factors [12]. Mapping studies for yellow pigment content (YPC) and yellow index (YI), in several biparental populations have identified QTLs on all wheat chromosomes (reviewed in Additional file 1: Table S1). The major QTL on the long arm of chromosome 7A, accounting for up to 60% of the phenotypic variation, was detected through all studies and attributed to allelic variations of the phytoene synthase (*Psy-A1*) gene [13–15]. Although there is an increased understanding of the mechanisms regulating carotenoid content and composition, only some carotenoid biosynthetic genes have been identified and cloned in wheat, such as phytoene synthase (*PSY*) [13, 16, 17], lycopene ε-cyclase (*LYCE*) [18, 19], carotene desaturase (*PDS*) and zeta-carotene desaturase (*ZDS*) [20], carotenoid β–hydroxylase (*BCH*) [21], lycopene β-cyclase (*LYCB*) [22].

As an alternative to classical linkage-based QTL mapping, the association mapping approach has received increased attention for detecting QTLs controlling complex traits [23]. One of the potential disadvantages of genome-wide association studies (GWAS) is the appearance of spurious marker-trait associations (false-positive associations) resulting from population structure and multiple testing of thousands of markers [24, 25]. Association mapping can be simplified for some traits by the “candidate gene approach”, that is testing SNPs within a candidate gene for a significant association with the trait [26].

The objectives of the current study were to: a) identify candidate carotenoid metabolic/catabolic genes in wheat by exploiting genomic resources and SNPs detected within the coding sequences of candidate genes; b) provide the precise map position of candidate genes on high-density SNP-based consensus maps; c) identify the genetic loci controlling yellow pigments by GWAS and candidate gene approaches using a tetraploid wheat collection coupled with the 90 K iSelect SNP genotyping array. The identification of genetic loci controlling yellow pigment accumulation/degradation will provide information on the genetic resources available to breeders to improve commercial and nutritional properties of wheat products, as well as the opportunity to develop functionally associated markers to be used in marker-assisted selection (MAS).

## Results

### Identification of carotenoid biosynthetic and catabolic genes of wheat

The *A. thaliana* isoprenoid pathways and respective genes from AtIPD (http://www.atipd.ethz.ch/) were used to identify and download the *Arabidopsis* gene sequences from the TAIR database (http://arabidopsis.org/). In order to isolate the wheat carotenoid sequences, the 24 cDNAs corresponding to all identified genes from *A. thaliana* database were used as query to extract sequences of *T. aestivum* and of the monocots *Brachypodium distachyon*, *O. sativa* and *Zea mays* (Table 1). The *in silico* analysis highlighted a lack of uniformity for acronyms and gene names/classifications used in literature between different plant species (e.g. the carotenoid β-ring hydroxylases is named *BCH* in *Arabidopsis*, *CRTR-B* or *HYD* in maize, and *BCH* or *HYD* in rice*, Brachypodium* and wheat). For simplicity, we used the gene nomenclature of *A. thaliana,* whose isoprenoid genes have been well characterized and reported in public metabolic pathway databases.

**Table 1:** Genebank entries of the carotenoid metabolic/catabolic pathways genes of *Arabidopsis thaliana, Triticum aestivum, Brachypodium distachyon, Oryza sativa* and *Zea mays*

Gene	Enzyme	*A. thaliana*	*T. aestivum*	*B. distachyon*	O. *sativa*	*Z. mais*
*PSY1*	Phytoene synthase 1	At5g17230	EF600063, EF600064	Bradi1g29590	AY445521	AY324431
*PSY2*	Phytoene synthase 2	At5g17230	DQ642445, DQ642441, BT009537	Bradi4g01100	Os12g43130	AY325302
*PSY3*	Phytoene synthase 3	-	Dibari et al. (2012)	Bradi4g37520	Os09g38320	DQ372936
*PDS*	Phytoene desaturase	At4g14210	FJ517553, BT009315	Bradi1g72400	AF049356	L39266
*Z-ISO*	cis-zeta-carotene isomerase	At1g10830	CV770956, CA501609^a^	Bradi4g08060	Os12g21710	BT036679, GRMZM2G011746
*ZDS*	Zeta-carotene desaturase	At3g04870	HQ703015, FJ169496, BT009332	Bradi1g54390	NP_001059145	AAD02462
*CRTISO*	Carotenoid isomerase	At1g06820	AK332627	Bradi1g67480	EF417892	FJ765413
*LYCB (LCYB)*	Lycopene β-cyclase	At3g10230	JN622196, BT009216	Bradi3g06600	Os02g09750	AY206862
*LYCE (LCYE)*	Lycopene ɛ-cyclase	At5g57030	EU649785, EU649786, EU649787	Bradi2g41890	BAC05562	NP_001146840
*BCH1 (CHYB1, HYD1)*	Carotenoid β-ring hydroxylase (β-hydroxylase 1)	At4g25700	JX171670, JX171671, JX171672, DR739690	Bradi1g76870	Os03g03370	GRMZM2G382534
*BCH2 (CHYB2, HYD2)*	Carotenoid β-ring hydroxylase (β-hydroxylase 2)	At5g52570	JX171673, JX171674, JX171675	Bradi5g19130	Os04g48880	GRMZM2G164318
*CYP97A3 (LUT5)*	Carotenoid β-ring hydroxylase (Cytochrome P450-type monooxygenase CYP97A3)	At1g31800	AK335215	Bradi3g55450	Os02g57290	GRMZM5G837869
*CYP97C1 (LUT1)*	Carotenoid ε ring -hydroxylase (Cytochrome P450-type monooxygenase CYP97C1))	At3g53130	BE499228^a^, CD862311^a^	Bradi3g32690	Os10g39930	GRMZM2G143202
*CCD7 (MAX3)*	Carotene (9,10) cleavage dioxygenase	At2g44990	BM137947^a^	Bradi5g17657	Os04g46470	GRMZM2G158657
*CCD8 (MAX4)*	Carotene (13,14) cleavage dioxygenase	At4g32810	BQ788859	Bradi2g49670	Os01g38580	GRMZM2G446858
*CCD1 (NC5)*	(5,6) (5′,6′) (9,10) (9′,10′) carotenoid cleavage dioxygenase	At3g63520	DR740786^a^	Bradi4g00330	Os12g44310	GRMZM2G057243
*CYP711A1 (MAX1)*	Cytochrome P450-type monooxygenase CYP711A1	At2g26170	CA742365	Bradi3g32690	JX566699	NM_001301569
*VDE*	Violaxanthin de-epoxidase	At1g08550	AF265294	Bradi5g07390	Os04g31040	EU956472
*ZEP (ABA1)*	Zeaxanthin epoxidase	At5g67030	AF384103	Bradi5g11750	Os04g37619	GRMZM2G127139
*NXS*	Neoxanthin synthase	At1g67080	CJ658959	Bradi2g01990	Os01g03750	GRMZM2G121747
*NCED4 (CCD4)*	9-cis-epoxycarotenoid dioxygenase	At4g19170	KP099105	Bradi3g52680	Os02g47510	GRMZM2G110192, GRMZM2G150363
*NCED9 (NC2; CCD9)*	9-cis-epoxycarotenoid dioxigenase 1	At1g78390	LC077864^a^	Bradi1g58580, XM_003561419	Os07g05940	GRMZM2G417954
*ABA2 (SDR)*	Short-chain alcohol dehydrogenase	At1g52340	EMS67256^a^, AK334048	Bradi1g04320	Os03g59610	GRMZM2G332976
*AAO3*	Abscisic aldehyde oxidase	At2g27150	EMS56969^a^, AK331622	Bradi1g52740	Os07g18120	NP_001105309

^a^partial EST sequence

**Fig. 2:**
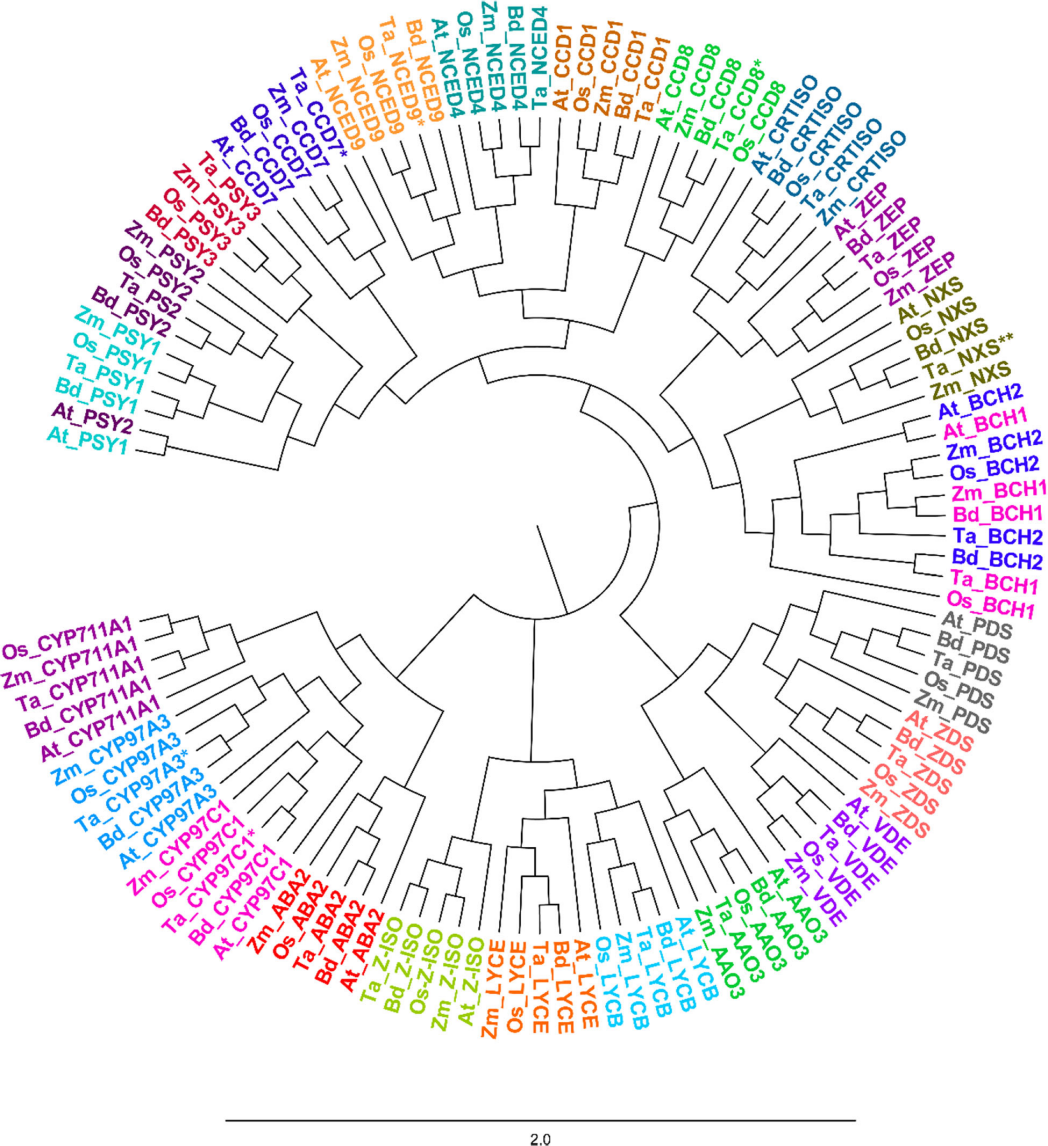
Phylogenetic tree of the carotenoid metabolic/catabolic genes from *Arabidopsis thaliana, Brachypodium disticum, Zea mays, Oryza sativa* and *Triticum aestivum*

The bootstrapped molecular phylogenetic tree (Fig. 2), based on 119 carotenoid cDNAs which correspond to orthologous sequences of the above-mentioned five plant species showed clear clustering of the orthologs by gene family. Additionally this analysis showed that these carotenoid genes are generally highly conserved between species, with the minimum sequence similarity being between *Arabidopsis* and *Brachypodium* for *NXS* (70%), and the maximum similarity observed between *Brachypodium* and rice for *CYP97C1* (89%). Sequence similarity helped to assign putative function to the identified wheat EST sequences. Table 1 lists the genebank entries of the carotenoid pathway genes of *Arabidopsis*, *Brachypodium*, rice, maize and wheat. The *PSY* gene family is tightly clustered based on the three paralogous genes, annotated as *PSY1*, *PSY2* and *PSY3*, while in eudicots only the presence of *PSY1* and *PSY2* homologs have been reported [17, 27]. The *BCH* characterization present in literature [21] was confirmed by the phylogenetic tree: *Ta_BCH1* clustered with *Zm_BCH2*, *Os_BCH2* and *Bd_BCH2*, while *Ta_BCH2* gene grouped with *Os_BCH1*.

The *in silico* gene expression analysis, using data from the publicly available Wheat 61 k GeneChip, revealed variation in transcription patterns for these carotenoid genes in a wide range of tissues and developmental stages in wheat (Additional file 2: Figure S1). Exploiting the PLEXdb database, the expression data was investigated to predict the genes’ impact on the final carotenoid content. In general, all carotenoid genes were found to be expressed to some degree during all developmental stages, with minimum expression levels of 3.53 and 4.51 RMA normalization for *Z-ISO* and *CCD7,* respectively, and maximum levels of 12.55 RMA normalization for *ZDS*. In particular, *PSY1*, *PSY2*, *PDS*, *ZDS*, *LYCB, CYP97C1*, *CCD1*, *VDE*, *ZEP* and *NCED4* showed elevated expression levels (values higher than the mean values ± 2 SD) in seedling leaf (phase 6) while *LYCE*, *BCH1* and *BCH2* genes exhibited high level of transcripts in anthers before anthesis (phase 10). *AAO3* showed higher levels of expression in reproductive tissues including immature pistil before anthesis. *ABA2* showed the highest expression during the caryopsis-embryo-endosperm growth (phase 11 to 13). Low expression values (mean values ± 2 SD) were detected for *LYCE* in roots, *CYP97C1* in anthers before anthesis, *CCD8* in 22 DAP endosperm stage and NXS in floral bracts before anthesis.

After the phylogenetic analysis, a BLASTn analysis (based on percentage identity) was performed between the 24 wheat carotenoid genes and the entire wheat SNP dataset [28], which provides a marker coverage of about 85% of the genome. A total of 75 SNP markers corresponding to the 19 carotenoid gene sequences were identified, with several genes containing multiple SNPs (Table 2). No SNP markers were identified within the *Z-ISO*, *CCD7*, *CCD8, CYP711A1* and *NXS* genes. Twenty-two and 32 SNP markers were located on the consensus durum [29] and bread wheat maps [28], respectively. This enabled us to assign genes to chromosomes groups; the *CRTISO* genes were mapped on chromosome group 1; *BCH1* and *VDE* on homoeologous chromosome arms 2 L; *LCYE* on group 3; *PDS* on group 4; *PSY2*, *PSY3*, *CCD1* and *ABA2* on group 5; *LUT5* on group 6; *PSY1* and *AAO3* on chromosome arms 7 L.

**Table 2:** Chromosome localization of the identified wheat carotenoid biosynthetic/catabolic genes on the durum [29] and bread wheat [28] consensus maps and allele frequency in the tetraploid wheat collection of 233 genotypes

Gene	Enzyme	SNP name	SNP id		Wheat map position			Allele frequency	
					Chrom.	Durum map	Bread map	Whole collection	Durum sub-population
*PSY1*	Phytoene synthase 1	BS00022137_51	IWB6923	A/G	7AL	203.2	-	0.26–0.74	0.12–0.88
		BobWhite_c4483_603	IWB3406	A/G	7AL	-	226.1	monomorphic	monomorphic
		CAP7_c12398_110	IWB13781	T/G	7BL	200.1	164.2	failed	failed
		BS00010747_51	IWB6281	T/G	7BL	200.1	164.2	failed	failed
		BS00084631_51	IWB11376	T/G	7DL	-	-		
		Kukri_rep_c105287_311	IWB49080^aa^	T/C	7DL		208.1		
*PSY2*	Phytoene synthase 2	BobWhite_c339_247	IWB2660	G/T	5AS	-	15.6	0.83–0.17	0.84–0.16
		RAC875_c51670_117	IWB58766	C/T	5AS	-	15.6	0.77–0.23	0.87–0.13
		Excalibur_c2922_3416	IWB24805^a^	C/T	5AS	46.6	15.6	monomorphic	monomorphic
		Excalibur_c30273_138	IWB24947	C/T	5BS	-	19.7	0.29–0.71	0.13–0.87
		Kukri_c23694_370	IWB42850	A/G	5BS	-	19.7	0.75–0.25	0.87–0.13
		GENE-3207_610	IWB33289	C/T	5BS	-	19.7	0.72–0.28	0.87–0.13
		GENE-3207_134	IWB33287	A/G	5BS	-	19.7	failed	failed
		Excalibur_c3948_1315	IWB26051^a^	C/T	5BS	15.8	19.7	0.43–0.57	0.48–0.52
		GENE-3207_174	IWB33288	A/G	5DS	-	-		
*PSY3*	Phytoene synthase 3	CAP7_c7840_316	IWB14264	A/G	5 L	-	-	monomorphic	monomorphic
		BS00077855_51	IWB10965^a^	C/T	5AL	126.8	82.0	0.74–0.26	0.88–0.12
*PDS*	Phytoene desaturase	BobWhite_c3609_361	IWB2819	A/G	4AS	-	-	0.01-0.99	monomorphic
		Tdurum_contig11560_383	IWB67151	C/T	4BL	-	-	monomorphic	monomorphic
		IACX725	IWB36188	A/G	4BL	-	-	monomorphic	monomorphic
		Kukri_c20125_281	IWB42264^a^	C/T	4BL	83.1	75.6	0.81–0.19	0.90–0.10
		Ra_c72128_236	IWB52568	A/G	4DL	-	-		
*Z-ISO*	cis-zeta-carotene isomerase	-	-						
*ZDS*	Zeta-carotene desaturase	CAP11_c754_335	IWB13046	A/G	2AS	-	-	monomorphic	monomorphic
		JD_c52783_234	IWB37604	C/T	2BS	-	-	monomorphic	monomorphic
		Kukri_c23475_1485	IWB42817	A/G	2BS	-	-	monomorphic	monomorphic
		RFL_Contig3540_283	IWB64386	A/C	2BS	-	-	monomorphic	monomorphic
		Ra_c5594_569	IWB52265	A/G	2DS	-	-		
		D_contig22022_580	IWB16076	T/G	2DS	-	-		
*CRTISO (CISO)*	Carotenoid isomerase	IAAV5931	IWB35115	A/G	1AS	-	66.9	0.98–0.02	monomorphic
		IAAV2888	IWB34631^a^	A/G	1AS	43.3	66.3	0.12–0.88	monomorphic
		CAP11_c292_249	IWB12814	T/C	1BS	-	-	monomorphic	monomorphic
*LYCB (LCYB)*	Lycopene beta-cyclase	CAP7_rep_c12997_415	IWB14395	A/G	6S	-	-	monomorphic	monomorphic
		Excalibur_c9273_1271	IWB29407^a^	T/C	6DS	-	-		
*LYCE (LCYE)*	Lycopene epsilon-cyclase	wsnp_Ex_c48136_53140385	IWA4009	T/C	3AL	-	85.4	monomorphic	monomorphic
		RAC875_c2375_132	IWB55558	A/G	3AL	-	-	monomorphic	
		IAAV4785	IWB34923^a^	G/T	3AL	65.9	85.4	0.29–0.71	0.35–0.65
		Excalibur_c34554_312	IWB25473^a^	C/T	3BL	67.2	64.4	0.31–0.69	0.16–0.84
		D_contig37716_635	IWB16739	A/G	3DL	-	-		
		Kukri_c196_184	IWB42160	AC	3DL	-	-		
*BCH1 (CHYB1, HYD1)*	Carotenoid β-ring hydroxylase (β-hydroxylase 1)	BobWhite_c34273_67	IWB2683	A/nulli	2AL	133.3	-	0.81–0.19	monomorphic
		Tdurum_contig5114_319	IWB72154^a^	C/T	2AL	-	112.1	0.51–0.49	0.30–0.70
		RFL_Contig329_957	IWB64290	T/nulli	2BL	-	-	0.90–0.10	monomorphic
		RFL_Contig329_877	IWB64289	A/G	2BL	-	-	monomorphic	monomorphic
*BCH2 (CHYB2, HYD2)*	Carotenoid β-ring hydroxylase (β-hydroxylase 2)	Tdurum_contig12547_293	IWB67643	T/C	4BL, 4DL, 5AL	-	-	monomorphic	monomorphic
*CYP97A3 (LUT5)*	Carotenoid β-ring hydroxylase (Cytochrome P450-type monooxygenase CYP97A3)	wsnp_JD_c7795_8868122	IWA6182	C/T	6AL	-	138.3	0.04–0.96	0.01–0.99
		wsnp_BF291974A_Ta_2_1	IWA441^a^	C/T	6AL	122.7	138.0	0.58–0.42	0.55–0.45
		Tdurum_contig569_263	IWB72540	A/G	6BL	145.3	108.9	0.77–0.23	0.75–0.25
		CAP11_c816_470	IWB13062^a^	A/G	6BL	145.3	-	0.54–0.46	0.33–0.67
		GENE-3988_631	IWB33711	A/G	6BL	145.8	-	0.04–0.96	monomorphic
		BobWhite_c12032_371	IWB293	T/C	6DL	-	133.54		
*CYP97C1 (LUT1)*	Carotenoid ε ring -hydroxylase (Cytochrome P450-type monooxygenase CYP97C1)	Kukri_rep_c111979_282	IWB49532	T/C	1AL	-			
		Excalibur_c29401_543	IWB24832	A/G	1BL	-			
*CCD7 (MAX3)*	Carotene (9,10) cleavage dioxygenase	-	-						
*CCD8 (MAX4)*	(13,14) cleavage dioxygenase	-	-						
*CCD1 (NC5)*	(5,6) (5′,6′) (9,10) (9′,10′) carotenoid cleavage dioxygenase	wsnp_Ex_c6209_10838456	IWB4445	A/G	5AS	-	-		
		CAP11_c2357_97	IWB12774	T/C	5BS	-	-		
		TA004832-0873	IWB65889	A/G	5BS	0.7	8.7		
		BobWhite_rep_c53718_103	IWB5053	T/C	5DS	-	-		
*CYP711A1 (MAX1)*	Cytochrome P450-type monooxygenase CYP711A1	-	-			-	-		
*ZEP (ABA1)*	Zeaxanthin epoxidase	RAC875_c856_92	IWB60810	A/C	2DL	-	-		
		CAP7_c4349_243	IWB14103	A/G	2DL	-	-		
		D_GBF1XID01ASYXD_209	IWB18346	A/G	2DL	-	-		
*VDE*	Violaxanthin de-epoxidase	wsnp_Ex_c11728_18862431	IWA1533	A/G	2AL	-	-	0.87–0.13	monomorphic
		wsnp_Ex_rep_c69465_68405569	IWA5610	C/T	2BL	-	99.8	0.08–0.92	0.02–0.98
		wsnp_Ex_c39862_47046812	IWA3696	C/T	2BL	-	99.6	0.01–0.99	monomorphic
		wsnp_Ex_c9805_16183499	IWA4965^a^	C/T	2BL	94.2	99.8	0.07–0.93	0.02–0.98
		Kukri_c52435_163	IWB46254	A/G	2BL	-	-	monomorphic	monomorphic
*NXS*	Neoxanthin synthase	-	-						
*NCED4 (CCD4)*	9-cis-epoxycarotenoid dioxigenase 4	wsnp_RFL_Contig4424_5193532	IWA8592^a^	C/T	6AL	62.8	82.4	0.24–0.76	0.07–0.93
		CAP11_c4654_171	IWB12893	A/G	6DL	-	-		
*NCED9 (NC2; CCD9)*	9-cis-epoxycarotenoid dioxigenase	CAP11_c5699_107	IWB12966	T/C	6DL	-	-		
		CAP7_c6372_329	IWB14194	A/G	6DL	-	-		
*ABA2 (SDR)*	Short-chain alcohol dehydrogenase	Tdurum_contig13608_72	IWB68029	T/G	5AL	-	-	failed	failed
		RFL_Contig4520_749	IWB64707	A/G	5BL	157.5	137.1	0.70–0.30	0.54–0.46
		Tdurum_contig13608_195	IWB68027	T/C	5DL	-	-		
*AAO3*	Abscisic aldehyde oxidase	RAC875_c64451_465	IWB59875	C/T	7AL	180.3	-	0.76–0.24	0.70–0.30
		Kukri_c5789_1029	IWB46699	T/C	7AL	180.3	-	failed	failed
		Ku_c5789_1180	IWB39660	G/T	7BL	155.7	120.8	0.37–0.63	0.24–0.76
		Excalibur_rep_c112889_341	IWB30603	A/G	7DL	-	184.18		

^a^SNP comigrating or mapping in the same contig of the gene sequence

### Phenotypic variation for yellow pigment content and yellow index

The tetraploid wheat collection, including 233 accessions of modern and old durum cultivars, durum landraces, domesticated and wild tetraploid wheat accessions, was evaluated for yellow index (YI) in six environments, and for yellow pigment content (YPC) in two environments. The analysis of variance showed highly significant differences among genotypes in each environment; environments, genotypes and environment x genotype interaction were significant in the combined analysis across environments (not shown). Mean, range, and heritability estimates (h_B_^2^) for YPC and YI of the whole collection, and of the durum wheat sub-population in each trial are reported in Table 3. A normal frequency distribution (Additional file 3: Figure S2) was observed for both traits. Mean values of YI of the whole collection varied from 12.8 (F09) to 14.6 (V10), while mean values of the durum sub-population ranged from 13.3 (F09) to 15.3 (V10). The phenotypic variation in the whole collection (from 9.1 to 17.8) and in the durum sub-population (11.6–17.8) suggested that alleles for low and high YI were present in the *T. turgidum* subset of the collection. YPC in the whole collection ranged between 3.2 and 11.7 μg/g at F08, and between 2.4 and 12.6 μg/g at V09, with average values of 6.3 and 5.8 μg/g, respectively. The durum sub-population showed higher mean values than the whole collection. This would indicate that in recent decades durum wheat breeders have paid special attention to the selection of new cultivars with grain colour that will be of higher (commercial) value [30].

**Table 3:** Mean, range of variation, standard deviation (SD), coefficient of variation (CV) and heritability (h^2^_B_) in the whole collection and in the durum sub-population evaluated for yellow index (b*) and yellow pigment content (μg/g) in six and two environments, respectively

Trait	Environment	Whole collection					Durum sub-population				
		Mean	Range	SD	CV	h^2^_B_	Mean	Range	SD	CV	h^2^_B_
Yellow index (b*)											
	Foggia 2009	12.8	(9.2–17.1)	1.3	3.45	0.89	13.3	(10.5–17.1)	1.1	3.30	0.86
	Foggia 2012	13.8	(9.6–19.0)	1.6	3.87	0.90	14.6	(10.0–19.0)	1.5	3.40	0.9
	Valenzano 2010	14.6	(11.4–18.5)	1.5	2.93	0.93	15.3	(11.6–18.5)	1.2	2.98	0.88
	Valenzano 2012	14.3	(8.9–18.8)	1.6	3.32	0.89	15.0	(11.8–18.8)	1.2	3.10	0.86
	Valenzano 2013	14.2	(9.2–18.4)	1.7	2.91	0.94	15.2	(11.9–18.4)	1.4	2.85	0.91
	Valenzano 2014	13.4	(9.3–17.6)	1.6	3.46	0.92	14.3	(11.2–17.6)	1.3	3.40	0.87
	Mean	13.8	(9.1–17.8)	1.5	3.32		14.6	(11.6–17.8)	1.2	3.17	
Yellow pigment (μg/g)											
	Foggia 2008	6.3	(3.2–11.7)	1.7	8.47	0.91	7.1	(3.8–11.7)	1.6	7.73	0.89
	Valenzano 2009	5.8	(2.4–12.6)	1.8	7.19	0.95	6.7	(2.8–12.6)	1.8	6.78	0.93
	Mean	6.0	(3.1–12.2)	1.8	7.83		6.9	(3.2–12.2)	1.6	7.26	

Broad-sense heritability in the whole collection ranged from 0.89 to 0.94 for YI, and from 0.91 to 0.95 for YPC. The high heritability values and the correlation coefficients among environments for YI and YPC (Tables 4 and Additional file 4: Table S2) indicated that both traits were stable, and that the phenotypic expression was mainly due to genotypic effects. Highly significant (0.001P) and positive correlation (*r* = 0.89) was observed between YPC and YI mean values across environments.

**Table 4:** Regression analysis between carotenoid genes and yellow index and yellow pigment content in a tetraploid wheat collection evaluated in six and two environments, respectively

Gene	SNP id	Wheat map position			Yellow Index			Yellow Pigment Content		
		Chrom.	Durum map	Bread map	-log_10_(P)	Effect	R^2^	-log_10_(P)	Effect	R^2^
*PSY1*	IWB6923	7AL	203.2	-	9.2***	1.37	16.8	5.2***	1.30	9.9
*PSY2*	IWB2660	5AS	-	15.6	ns			ns		
	IWB58766	5AS	-	15.6	3.7**	0.83	5.9	ns		
	IWB24947	5BS	-	19.7	4.7***	0.93	7.9	ns		
	IWB42850	5BS	-	19.7	4.3**	0.90	7.0	ns		
	IWB33289	5BS	-	19.7	4.3**	0.88	7.0	ns		
*BCH1*	IWB2683	2AL	133.3	-	9.6***	1.49	16.00	6.7***	1.56	12.1
	IWB64290	2BL	-	-	ns			4.2**	1.63	7.4
*CYP97A3*	IWB72540	6BL	145.3	108.9	ns			ns		
	IWB13062	6BL	145.3	-	5.8***	0.93	9.7	6.8***	1.25	12.4
*VDE*	IWA1533	2AL	-	-	9.4***	1.71	15.8	6.7***	1.97	12.1
*ABA2*	IWB64707	5BL	157.5	137.1	9.2***	-1.34	16.3	7.7***	1.51	14.8
*AAO3*	IWB59875	7AL	180.3	-	3.8**	-0.86	6.5	ns		
	IWB39660	7BL	155.7	120.8	3.8**	0.79	6.4	ns		

**and *** = significant at *P >* 0.01and *P >* 0.001, respectively, using the Bonferroni threshold (P/28) to control for multiple testing; ns = not significant; R^2^ = Phenotypic variation explained by the QTL (%)

### Association of carotenoid genes to yellow pigments

Out of 24 carotenoid candidate genes, 17 showed no SNPs in the coding sequences, failed in the array analysis, or had an allele frequency lower than 0.10 (Table 2) in the wheat collection. These genes were therefore removed from the Marker Trait Association (MTA) analysis. Seven candidate genes (*PSY1, PSY2, BCH1, CYP97A3, VDE, ABA2* and *AAO3*) had between 1 to 5 SNPs, and a linear regression analysis was carried out between each SNP, and YPC and YI (Table 4). Except for *BCH1* on 2BL, one or more SNPs of each candidate gene mapped onto one or both homeologous chromosomes were found to be significantly associated to YI, indicating their involvement in the yellow pigment biosynthesis or catabolism. *PSY1, BCH1, CYP97A3, VDE* and *ABA2* were also significantly associated to YPC. The phenotypic variation (R^2^) explained by each of these markers varied from 5.9 to 16.3% for YI and from 7.4 to 14.8% for YPC. The estimated allelic effects for each marker ranged from −1.34 to 1.79 units for YI, and from 1.25 to 1.97 μg/g for YPC.

### Detection of QTLs by GWAS

The wheat collection had been genotyped using the 90 K iSelect array. After excluding SNPs on the basis described in the methods, 13,639 SNPs in the whole collection and 9,863 SNPs in the durum sub-population were used for the association analysis. All of these SNPs have locations on the durum consensus map [29]. MTAs were initially calculated by linear regression analysis (GLM) and by three more statistical models (GLM + PCs, MLM + K, MLM + K + PCs) taking into account the confounding effects of population structure and relative kinship to minimize the occurrence of false-positive associations. In general, unsurprisingly the number of significant MTAs with GLM and GLM + PCs was much higher than with MLM + K and MLM + K + PCs (Additional file 5: Table S3). The strong deviation of the observed -log_10_(*P*) values from the expected distribution (see Q-Q plots in Additional file 6: Figure S3) and the high number of significant MTAs clearly indicated the detection of numerous false-positives by GLM and GLM + PCs models. Observed *P* values were closer to expected distribution incorporating the K matrix only or the K matrix and the PCs into a MLM, providing more confidence in the associations for YI and YPC detected using this model. The MLM + K and MLM + K + PCs models gave similar results; to minimize possible false-positives we decided to focus on the results generated by the MLM + K + PCs model.

GWAS based on mean values of YI across environments detected nine significant QTLs in the whole collection, and five QTLs in the durum sub-population (Table 5). The QTLs identified in the analysis of the whole population were on chromosomes 4A, 4B (two), 5B, 7A (four) and 7B. The QTLs identified in the durum sub-population were on 4B (two) and 7A (three). Four QTLs (two on 4B and two on 7A) were identical in both analysis (the whole collection and in the durum sub-population). Out of nine significant QTLs for YI across environments, the QTL on 7A at 102.3 cM fulfilled the more stringent FDR criteria. The phenotypic variation (R^2^) for each of these markers varied from 4.8 to 6.1% in the whole collection and from 10.1 to 18.4% in the durum sub-population. The estimated allelic effects for each marker ranged from −1.25 to 1.33 units.

**Table 5:** SNP markers significantly associated (−log10(*p*) ≥ 3) with yellow index identified by GWAS (model MLM + K + PCs) in the whole tetraploid wheat collection and in the durum sub-population evaluated in six environments (F09, V10, V12, F12, V13, V14)

SNP marker	SNP allele	Chrom	Position cM	Whole collection									Durum sub-population								
				F09	V10	V12	F12	V13	V14	Mean	R^2^ (%)	Effect	F09	V10	V12	F12	V13	V14	Mean	R^2^ (%)	Effect
IWB73278	C/T	1B	12.8	-	-	3	-	-	-	-			-	-	-	-	-	-	-		
IWB4839	C/T	1B	150.9	3.4	-	-	-	-	-	-			-	-	-	-	-	-	-		
IWB70428	A/C	2A	101.5	3.4	3	-	-	-	-	-			-	-	-	-	-	-	-		
IWB42586	C/T	2A	176.5	-	-	-	-	4.2	-	-			-	-	-	-	-	-	-		
IWB55230	C/T	2A	196.5	-	-	-	-	-	-	-			-	-	3.2	-	-	-	-		
IWB45885	C/T	2B	14.5	-	3.2	-	-	-	3.1	-			-	-	-	-	-	-	-		
IWB1756	A/G	3B	33.2	3.2	-	-	-	-	-	-			-	-	-	-	-	-	-		
IWB58482	C/T	3B	160.1	-	-	-	3.2	-	-	-			-	-	-	-	-	-	-		
IWB43375	A/G	4A	80.5	-	3.4	3.3	-	-	-	3.0	4.8	−0.98	-	-	-	-	-	-	-		
IWB58319	A/G	4B	17.7	-	3.1	3.3	-	-	-	3.0	4.9	−0.58	-	3.6	-	-	-	3.1	3.3	10.1	−0.8
IWB72011	C/T	4B	43.9	-	3.4	-	-	3.1	-	3.4	5.5	0.72	-	3	-	-	3	3.7	3.3	11.1	1.33
IWB72977	A/G	5A	113.7	-	-	-	-	-	3.1	-				-	-	-	-	-	-		
IWB71274	A/C	5B	44	-	-	-	-	-	3.4	-				-	-	-	-	-	-		
IWB43483	A/G	5B	120.1	-	3.5	3	-	-	-	3.1	5.1	−0.72		-	-	-	-	-	-		
IWB62049	G/T	5B	167.5	-	-	-	-	3.2	-	-				-	-	-	-	-	-		
IWB14365	A/G	6A	93.4	-	-	-	3.2	-	-	-				-	-	3.1	-	-	-		
IWB73296	A/G	6A	115.3	-	-	-	3.2	-	-	-				-	-	-	-	-	-		
IWB68640	G/T	7A	14.1	3.2	-	-	-	3.2	4.6	3.5	5.7	0.81		-	-	-	-	-	-		
IWB8374	A/G	7A	61.6	3.5	3.1	-	3.4	-	-	-			3.9	-	3.2	3.2	-	3.5	3.7	12.6	1.08
IWB72567	C/T	7A	102.3	4.1	5.5		4.1	3.6	-	3.5	6.2	−0.74	3.4	4.9	4	4	4.5	3.5	5	18.4	−1.25
IWB20381	C/T	7A	168.8	3.1	-	-	-	-	-	-			-	-	-	-	-	-	-		
IWB59875	C/T	7A	180.3	-	5.4	3.1	4.2	3.2	3.1	3.7	6.1	0.83	-	4.3	3.1	4	-	-	3.8	12.2	0.92
IWB49295	A/G	7A	203.4	-	4.2	-	-	3.1	3.9	3.5	5.8	−0.78	-	-	-	-	-	-	-		
IWB72335	A/C	7B	58.3	-	-	-	-	3.1	-	-			-	-	-	-	-	-	-		
IWB9496	A/G	7B	185.2	-	-	-	-	-	4	3.5	5.8	−0.72	-	-	-	-	-	-	-		

- = not significant

R^2^ = Phenotypic variation explained by the QTL (%)

Chromosome and map position from Maccaferri [29] and -log10(*p*) values are reported for each marker in each environment and in the mean of the environments. Phenotypic variation (R_2_) and additive effect are reported only for markers significant in the mean of all six environments

GWAS based on mean values of YPC over two environments (Table 6) detected three significant QTLs on chromosomes 4B (one) and 7A (two) both in the whole collection and in the durum sub-population, and one additional QTL on 4B (position 43.9 cM) in the durum sub-population. The QTL on 7A associated to the SNP marker IWB49295 located in the *Psy-A1* coding sequence was consistent in both the whole collection and the durum sub-population. Out of four significant QTLs for YPC across environments in the durum sub-population, the QTL on 7A at 102.3 cM passed the FDR criteria. The phenotypic variation (R^2^) explained for each of these markers varied from 5.3 to 22.1%, while the allelic effects for YPC ranged from −1.90 to 1.79 μg/g.

**Table 6:** SNP markers significantly associated (−log10(*P*) ≥ 3) with yellow pigment content identified by GWAS (model MLM + K + PCs) in the whole tetraploid wheat collection and in the durum sub-population evaluated in two environments (F08, V09)

SNP marker	SNP allele	Chrom	Position cM	Whole collection						Durum subpopulation					
				Allele frequency	F08	V09	Mean	R^2^ (%)	Effect	Allele frequency	F08	V09	Mean	R^2^ (%)	Effect
IWB9815	A/G	3B	93.8	0.15–0.85	3.4	-	-				-	-	-		
IWB58319	A/G	4B	17.7	0.50–0.50	-	3.8	3.0	5.3	−0.84	0.55–0.45	-	3.8	3.3	10.9	−1.17
IWB72011	C/T	4B	43.9	0.67–0.33	-	-	-			0.84–0.16	-	3.3	3.0	10.9	1.79
IWB68046	A/G	5A	84.2	0.84–0.16	3.0	-	-				-	-	-		
IWB57337	A/G	5B	53.4	0.70–0.30	-	3.1	-				-	-	-		
IWB72567	C/T	7A	102.3	0.53–0.47	6.0	5.5	6.1	13.6	−1.54	0.28–0.72	5.7	4.9	5.4	22.1	−1.90
IWB49295	A/G	7A	203.4	0.34–0–66	-	3.8	4.0	7.2	−1,10	0.19–0.81	-	3.2	3.2	10.4	−1.43

- = not significant

R^2^ = Phenotypic variation explained by the QTL (%)

Chromosome and map position from Maccaferri [29] and -log10(*P*) value are reported for each marker in each environment and in the mean of the environments. Phenotypic variation (R^2^) and additive effect are reported only for markers significant in the mean of all two environments

To investigate the environmental variations on detection of significant QTLs by GWAS, the MTA analysis was carried out on the mean value over replicates for each of the six environments for YI and for each of the two environments for YPC (Tables 5 and 6). A high QTL-to-environment variation was observed for both traits as we identified 17 QTLs specific in single environments vs. common QTLs across environments. Considering the GWAS for YI in the whole collection, a minimum of 5 QTLs were detected at V12 and a maximum of 11 QTLs at V10. Eleven different QTLs were only identified in one environment, 7 in two environments, 4 in three environments, 1 in four environments and only 1 in five environments. Notably, no QTL was detected in all six environments. Genotype x environment (QTL x E) interaction was lower in the durum sub-population: 2 QTLs were detected in two environments, 3 in three environments, 1 in four environments and 1 in all six environments. The same trend was observed for YPC: 5 QTLs were identified in only one environment and 1 in both examined environments in the whole collection; out of 4 QTLs detected in the durum sub-population, 3 QTLs were consistent in one environment and 1 in both environments.

## Discussion

### Identification and mapping of carotenoid genes in the wheat genome

The carotenoid biosynthetic pathway has been extensively studied in model plants and crop species due to their important roles in both development and photosynthesis [2], and their beneficial effects on human health [5]. The wheat genome has still not been completely sequenced due to its huge size and complexity, and the knowledge of metabolic and catabolic pathway of carotenoid compounds remains incomplete.

Comparative genomic analysis across different taxa allowed to transfer functional information from well-characterized model organisms, such as *Arabidopsis,* rice and *Brachypodium*, to another less-studied taxon, like wheat. This has been beneficial for *BCH1*, *BCH2*, *CYP97C1*, *CCD7*, *CCD1*, *NCED9* and *CCD7* genes, many of which have been well characterized in rice, *Brachypodium* and *Arabidopsis*, but few of which have been studied in wheat. All the orthologues clustered by gene on the phylogenetic tree, sharing common conserved motifs in cDNA sequences. Unsuprisingly, the phylogenetic analysis revealed that the dicotyledonous *PSY1* and *PSY2* groups were more distantly related to those of the monocotyledonous groups, thus supporting the assumption that a single duplication event of the ancestor genes occurred before the divergence of the grass subfamilies [17, 27]. Differential duplication events took place in the *BCH* clade. A separation of the *Arabidopsis BCH* paralogs suggested the same time frame as the other genes for functional diversification [21], but an unexpected separation occurred prior to the main grass subfamily divergence for rice *BCH1*. Further studies on the gene structure and intron-exon size facilitate a better understanding of the *BCH* group. The *in silico* expression analysis of the carotenoid candidate genes included in the present study in a wide range of tissues and developmental stages showed that many of these genes had similar expression profiles. Additionally we observed that sometimes one or more genes were virtually unexpressed (such as *Z-ISO* and *CCD7*) or highly expressed (such as *ZDS*) in all the thirteen tissues/stages (Additional file 2: Figure S1). *LYCE*, *BCH1*, *BCH2*, *CYP97A3* and *ABA* genes exhibited high expression levels in the anthers prior to anthesis and in kernel tissues, indicating their potential involvement in kernel carotenoids accumulation.

**Fig. 3:**
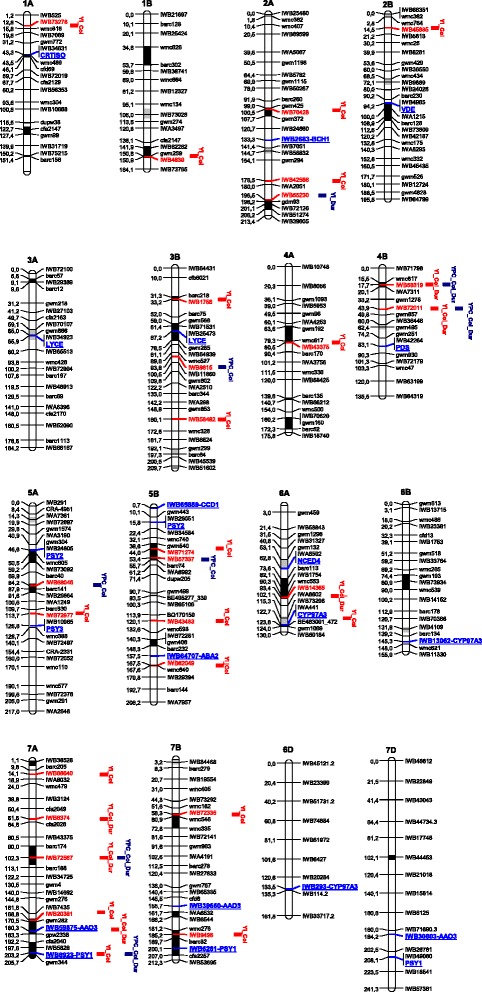
Schematic representation of wheat genome chromosomes. The map is a representation of A and B genome chromosomes of the durum consensus linkage map [29] and of D chromosomes of the consensus bread wheat map [28], with map positions of carotenoid candidate genes and QTLs for yellow index and yellow pigment content. Each chromosome map is represented by the first and the last SNP marker, and by a SNP marker every about 20 cM. SSR markers have been also inserted every about 20 cM to compare the consensus SNP map with published SSR-based maps. Markers are indicated on the right side and cM distances on the left side of the bar. Solid regions of the chromosome bars indicate regions identified as being significantly associated with YI and YPC in published QTL biparental mapping populations (black regions in at least two different populations, grey regions in one population). QTLs are represented by bars on the right of each chromosome bar. QTL names indicate the trait (YI for yellow index and YPC for yellow pigment content) and the population in which the QTL was detected (Col = whole collection and Dur = durum sub-population); the closest SNP marker is indicated in red. Carotenoid genes are indicated after the corresponding SNP located in the gene sequence (in blue) or in the same map position of the co-migrating SNP marker located in the same contig

With the objectives of both characterizing the carotenoid genes and investigating their relationships with the amber colour of grain and flour of wheat, we analyzed a tetraploid wheat collection with the recently developed genotyping array including 81,587 gene-associated SNPs [28]. The BLASTn analysis of the entire SNP dataset against the carotenoid gene sequences allowed to identifying 1–7 SNPs in the coding sequences of 19 out of 24 examined carotenoid candidate genes (Table 1). In many cases, at least one SNP was identified for each of the three homeologous genes present in the wheat genomes (*PSY1, PSY2, PDS, ZDS, LYCE, CYP97A3, CCD1, ABA2* and *AAO3*). The recent availability of the high-resolution consensus map of durum [29] and common wheat [28] allowed us to determine the precise map position of most of the carotenoid genes (Table 1 and Fig. 3). The chromosomal location of 13 carotenoid genes determined by our strategy was consistent with results reported by Crawford and Francki (2013) [19], who identified the chromosomal locations based on survey sequence from the International Wheat Genome Sequencing Consortium (http://www.wheatgenome.org/). Map positions of a few carotenoid gene are reported in chromosome intervals as long as 5–20 cM in different SSR-based maps, such as *PSY1* and *PSY2* [16] and *LYCE* [31]. The carotenoid genes are distributed on 14 of the 21 chromosomes of bread wheat, and the identification of functional markers and map position can be particularly useful for breeders in MAS programs.

### Association of carotenoid genes to yellow pigments

The allele frequency of SNP markers corresponding to carotenoid genes were found to be very variable in the examined wheat collection (Table 2). Several of these SNPs were either monomorphic, or had a MAF < 10% and therefore considered to be rare alleles. *PSY1, PSY2, BCH1, CYP97A3, VDE, ABA2* and *AAO3* were significantly associated to YPC and YI (Table 4), and this validated previous results obtained by using biparental mapping populations for *PSY1* [15, 16], *LYCE* [19, 31] and *AAO3* [32]. The association of *PSY2, BCH1, CYP97A3, VDE* and *ABA2* genes with YI and YPC is novel, and indicated that the SNP markers identified within the carotenoid gene sequences can represent a resource for developing genetic markers for use in marker assisted breeding.

Ten carotenoid metabolic/catabolic genes were mapped in corresponding chromosome regions with QTLs detected in the current work and/or in previous QTL studies (see review in Additional file 1: Table S1 and Fig. 3) indicating possible relations between candidate genes and grain colour-related traits. Six genes (*CRTISO*, *VDE*, *LYCE*, *PSY2*, *CYP97A3* and *PSY1*) are directly involved in the biosynthesis of carotenoid compounds [2]. Interestingly, the catabolic genes *NCED9*, *ABA2* and *AAO3*, involved in the carotenoid cleavage to process violaxanthin and neoxanthin into abscisic acid, were located in chromosome regions influencing YPC [32–34]. These data are consistent with findings in other plant species such as *Arabidopsis* and maize [35, 36], demonstrating that carotenoid degradation is important in determining total carotenoid accumulation.

### QTLs detected by GWAS and comparison with previous studies

In addition to the candidate gene approach, we conducted a GWAS by using the GLM and the MLM models taking into account the confounding effect of population structure and the relative kinship. Q-Q plots clearly indicated the MLM (K + PCs) as the most suitable model for the GWAS of YPC and YI, thus confirming other results of GWAS on quantitative traits carried out on crop plants [37]. Several QTLs for YPC and YI, distributed on 12 of the 14 chromosomes of durum wheat, were detected (Tables 4 and 5 and Fig. 3). Four stable QTLs on 4B (two) and 7A (two) were associated with both YI and YPC, explaining the significant and positive correlation between the two colour-related traits found in the present and previous studies [38–40]. The higher number of QTLs for YI indicated that yellow pigments of wheat kernels are synthesized by different biochemical pathways, including that for the carotenoids, which interact in some way with the accumulation of carotenoids, such as polyphenol oxidase (PPO), lipoxygenase (LPX) and other carotenoid oxidative enzymes [10, 11]. In addition, it is possible that the wider variability of the entire wheat collection is determined by more genes influencing colour-related traits, and that some yellow pigment genes have been fixed during the breeding programs for grain colour improvement and therefore not detected in the durum sub-population.

Several studies on QTL mapping of yellow pigments in wheat have been published during the past two decades. A detailed list of QTLs detected in 26 peer-reviewed papers is reported in Additional file 1: Table S1 and the majority of them are illustrated in Fig. 3. Except chromosome 1D, QTLs for yellow pigments were detected on all wheat chromosomes. Results of QTL mapping studies indicated many differences in the number and map position of QTLs detected in the different experiments. This may be attributed to a high number of effective genes underlying QTLs coupled with: a) different contributions from parental genotypes of mapping populations; b) QTL x environment interactions; c) differences in the carotenoid extraction procedures and colour measurement, therefore different gene-to-trait associations revealed; d) marker density of linkage maps used in QTL analyses; e) differences in the statistical procedures used for QTL detection and threshold used for the statistical significance of MTAs.

While many of the QTLs for YI and YPC identified in the current study had been described previously (see Fig. 3 for a detailed comparison), 11 QTLs detected on 1AS, 2AL, 2BS, 3BL (two), 4BS, 5AS, 5BS (two) and 7AS (two) were new. Four of these QTLs were detected in more than one environment (Table 5 and Table 6), indicating that some wheat accessions of the examined collection possess new stable alleles potentially useful for improving colour and nutritional value of wheat grain. Additionally 16 QTLs detected in the present study (on chromosome arms 1BL, 2AL (two), 3BS, 4AL, 4BS, 5AL, 5BL, 6AL (two), 7AL (five), 7BL (two)) validated QTLs previously detected in different genetic backgrounds. Therefore these QTLs can be considered as stable and useful for MAS in breeding programs.

### Genotype x environment interaction and QTL detection

With the aim to investigate if the results of GWAS were affected by environmental fluctuations, we conducted replicated trials for YI and YPC in six and two environments, respectively. Comparing the GWAS results, large variations in the number and type of QTLs were observed for both traits in different environments, thus confirming the existence of genotype x environment interaction effects as indicated by the variance analysis. Stable associations for YI in at least three over six environments in the whole collection were detected for five QTLs corresponding to one genomic region on chromosomes 5B, and four regions on 7A. In many cases, the SNP-trait associations were environment-specific, as 11 QTLs were consistent only in one environment and 7 in two environments. The same trend was observed for YPC evaluated in two environments. Although the high values of heritability (from 0.89 to 0.94 for YI and from 0.91 to 0.95 for YP) in open field trials, the complexity of the genetic basis of the studied traits tends to confound the interpretation of GWAS results. These findings are consistent with results obtained by association mapping and QTL linkage analyses experiments on complex traits with far lower heritability such as yield and yield components [41, 42]. The present study suggests that QTL analysis for agronomically important “true” quantitative traits should be always conducted in a plurality of environments with different soil and climatic conditions. Finally, the need to evaluate and take into account the G x E interaction is important in breeding programs to identify genotypes adapted in a wide range of environments.

### Comparison between simple regression and MLM analysis for QTL detection

The SNPs located in the gene sequences *PSY1, PSY2, BCH1, CYP97A3, VDE* and *ABA2* were significantly associated to YI and YPC by regression analysis but not by GWAS analysis. Only the SNP marker IWB59875 located in the coding sequence of the abscisic aldehyde oxidase (*AAO3*) on chromosome arm 7AL was consistent by both MTA analyses. The *PSY1, PSY2, CYP97A3* and *VDE* genes were mapped on chromosome regions corresponding to QTLs for YI and YPC detected in the current study by GWAS or by previous studies using biparental mapping populations (see Fig. 3). *NCED, CRTISO* and *LYCE*, which were excluded from the regression analysis as they had allele frequencies lower than 0.05, were also mapped in chromosome regions corresponding to QTLs for YI and YPC. The same results were obtained by Zhao [43], who detected several SNPs near height-controlling genes consistent only by the naïve approach, and suggested that mapping populations derived from crosses between genetically distant parents could be needed to complement GWAS to reduce the rate of both false positives and false negatives. It is well known that GWAS carried out by the GLM model generally gives a high number of false-positives [44], and that it is necessary to take into account the confounding effect of population structure and relatedness among individual to control the overall probability of type I error [37]. However, reducing the number of false positives may lead to increasing the number of false negatives, and in some situation ignoring most of the important findings on the genetics and physiology of the traits of interest [45]. The combination of population genetic models and molecular biological knowledge into new QTL detection methods has been recently proposed to increase statistical power of GWAS in human and agricultural research, as to reduce the overall probability of type II error (false-negative associations), and incorporate biological context in GWAS results [46].

## Conclusions

GWAS analysis in wheat collections can contribute to validate QTLs previously detected in biparental populations and to unravel new QTLs for colour-related traits. The MLM models can reduce the number of false positives, while the candidate gene approach can contribute to reduce the number of false negatives. However, GWAS analysis should be carried out on phenotypic data measured in more environments to detecting stable QTLs and determining the genotype x environment interactions that tend to confound the interpretation of MTAs and the genetic dissection even of quantitative traits with high heritability values. The availability of markers within the coding sequences of candidate genes can allow to elucidating the mechanism of carotenoid accumulation in the wheat kernels and to exploiting the genetic variability of colour-related traits for the nutritional and commercial improvement of end-finished products of wheat.

## Methods

### Plant materials and phenotypic evaluation

A collection of 233 accessions of tetraploid wheat (*Triticum turgidum* L., 2*n =* 4× = 28; AABB genome) was grown at Valenzano (Bari, southern Italy, 41°02′46″N, 16°53′09″E, altitude 118 m a.s.l., annual average rainfall 586 mm, average temperature 15,7 °C) for five years (2009, 2010, 2012, 2013 and 2014, hereafter reported as V09, V10, V12, V13, V14) and at Foggia (southern Italy, 41°32′11″N, 15°43′01″E, altitude 60 m a.s.l., annual average rainfall 469 mm, average temperature 15,4 °C) for three years (2008, 2009 and 2012, hereafter reported as F08, F09 and F12). The panel included accessions of seven *T. turgidum* subspecies: *durum* (124 accessions), *durum* var. *ethiopicum* (10), *turanicum* (20), *polonicum* (19), *turgidum* (16), *carthlicum* (14), *dicoccum* (18) and *dicoccoides* (12). The wheat collection has been extensively characterized in terms of genetic diversity and population structure [47], and has been used for the association mapping of loci controlling the resistance to stem rust [48] and β-glucan content [49]. A detailed list of genotypes (number/name, year of release, country, pedigree) is provided by Laidò [47]. A randomized complete block design with three replications was used with plots consisting of 1-m rows, 30 cm apart, with 50 germinating seeds per plot. During the growing season, standard cultivation practices were used. Grain samples were ground in a laboratory mill with a 1 mm sieve and the resulting whole flour stored at −4 °C for a maximum of 24 h before analysis. The determination of YPC was made according to AACC Approved Method 14–50 [50] with slight modifications as described by Fares [51]. YI was determined using the reflectance colorimeter Chroma Meter CR-300 (Minolta) and the “b*” value indicating the yellow intensity was used in subsequent analysis.

### DNA extraction and SNP genotyping

Genomic DNA was isolated from freeze-dried leaf tissue following the protocol by Dellaporta [52]. A total of 50 ng/μL of genomic DNA of each accession was analyzed with the wheat 90 K iSelect array [28]. Genotyping was performed at TraitGenetics GmbH (http://www.traitgenetics.de) following the manufacturer’s recommendations as described in Akhunov [53]. The genotyping assays were carried out to the Illumina iScan reader and performed using Genome Studio software version 2011.1.

### Identification of putative carotenoid biosynthetic and catabolic gene sequences

The *Arabidopsis thaliana* isoprenoid pathways and respective genes from AtIPD (http://www.atipd.ethz.ch/) were used to identify and download from the TAIR database (http://arabidopsis.org/) the cDNA sequences involved in the carotenoid biosynthetic and catabolic pathway. Orthologous genes for *Brachypodium distachyon*, *Oryza sativa*, *Zea mays* and *Triticum aestivum* were retrieved from the UniGene Cluster database at NCBI (https://www.ncbi.nlm.nih.gov/) by carotenoid keyword searching. Phylogenetic analysis was carried out using the Neighbor-Joining method and a 1000 replication bootstrap test for significance [54]. In order to denote the plant species, a two-letter prefix was placed before each gene symbol considering *At* for *A. thaliana*, *Bd* for *B. distachyon*, *Os* for *O. sativa*, *Zm* for *Z. mays* and *Ta* for *T. aestivum*. The alignment of each cDNA was performed via Mega4 software [55]. The tree was generated with ClustalW2 (http://www.ebi.ac.uk/Tools/phylogeny/) and depicted with the program FigTree (http://tree.bio.ed.ac.uk/software/figtree/).

Wheat carotenoid gene sequences were blasted against the available dataset of SNP marker sequences reported by Wang [28], and markers aligned with 80% (IUM) identity were considered as markers within the coding sequences of the carotenoid genes. The BLASTn analysis was extended to contigs assembled in the chromosome survey-sequencing project (http://wheat-urgi.versailles.inra.fr/Seq-Repository) to identify additional SNPs flanking the carotenoid genes. All the retrieved wheat carotenoid cDNA sequences were blasted against the Wheat 61 k GeneChip in PLEXdb database (http://www.plantgdb.org) for obtaining information on carotenoid gene expression variation in different development phases.

### Statistical analysis and QTL detection

Each year-location combination was considered as an environment, and analysis of variance was carried out using the standard procedure with the software MSTAT-C. Genetic variance (σ^2^_G_), environmental variance (σ^2^_E_) and broad-sense heritability (h^2^_B_ = σ^2^_G_/(σ^2^_G +_ σ^2^_E_ + σ^2^_GxE_) were obtained using the variance component estimates.

Pearson correlation coefficients were calculated between YPC and YI. Details about genetic diversity and population structure of the tetraploid wheat collection as investigated with SSR and DArT markers are provided by Laidò et al. [47], and with SNP markers by Marcotuli et al. [49]. Using Bayesian clustering (K = 2), both sets of molecular markers distinguished the durum cultivars from the other tetraploid subspecies accessions; accordingly, GWAS was conducted on the whole collection and on the 124 durum varieties (hereafter referred to as durum sub-population). Mean values across replicates and mean values across replicates and years of YI and YPC were used in the GWAS for each environment and over environments, respectively. Prior to GWAS, markers that had >10% missing data points and markers with a minimum allele frequency (MAF) of less than 10% were removed from the data matrix. Unmapped markers on the consensus durum wheat map [29] were not used for association analysis. GWAS was carried out using TASSEL v.5 (http://www.maizegenetics.net) with and without correction for population structure. Associations between SNP markers and YPC and YI were calculated using the following models: a) simple regression analysis (general linear model, GLM); b) GLM including population structure as a covariate by using the Q-matrix derived from the principal component analysis (PCA) as implemented in TASSEL (GLM + PCs); c) mixed linear model (MLM) based on the kinship-matrix (MLM + K); d) mixed linear model based on both Q-matrix and K-matrix (MLM + K + PCs). The statistical models used in the present GWAS were extensively reviewed by Astle and Balding [25] considering the most widely used statistical approaches for controlling the confounding effects of population structure. The most appropriate GWAS method was chosen by inspection Q-Q plots and Manhattan plots for evidence of *P* value inflation. A marker-trait association was considered significant when one or more markers were associated with YPC or YI at threshold –log_10_(*P*) ≥ 3.0 determined by the modified Bonferroni correction as implemented in Genstat (GenStat, 2003). A false discovery rate (FDR) at 0.05P was calculated by the q-value package in R software [56]. For the associations between carotenoid candidate genes and YPC and YI, the conservative Bonferroni correction for multiple testing was calculated by dividing *P <* 0.01 with the number of markers used in the analysis. Chromosome localization and map position of SNP markers were derived from the high-density linkage maps described by Maccaferri [29] for durum wheat and by Wang [28] for common wheat used as reference maps.

## Additional files

Additional file 1: Table S1. List of detected QTLs for yellow index and/or yellow pigment content in wheat. (DOCX 18 kb)
Additional file 2: Figure S1. Expression analysis from PLEXdb database of all key genes in carotenoid biosynthesis. (DOCX 83 kb)
Additional file 3: Figure S2. Frequency distributions of yellow index and yellow pigment content. (DOCX 28 kb)
Additional file 4: Table S2. Correlation coefficients of yellow pigment content and of yellow index. (DOCX 12 kb)
Additional file 5: Table S3. Number of significant marker-trait associations detected by four GWAS models. (DOCX 13 kb)
Additional file 6: Figure S3. Genome-wide association analysis for yellow index and yellow pigment content: Q-Q plots. (DOCX 1809 kb)

## Abbreviations

At: *Arabidopsis thaliana*
Bd: *Brachypodium distachyon*
Os: *Oryza sativa*
QTLs: Quantitative trait loci
SNPs: Single nucleotide polymorphisms
Ta: *Triticum aestivum*
YI: Yellow index
YPC: Yellow pigment content
Zm: *Zea mays*
