# Supplementary material for: The carotenoid biosynthetic and catabolic genes in wheat and their association with yellow pigments
Source: BMC Genomics. 2017 Jan 31;18:122. doi: 10.1186/s12864-016-3395-6 (PMC5286776; doi:10.1186/s12864-016-3395-6)
Supplement: Additional file 1: Table S1. — List of detected QTLs for yellow index and/or yellow pigment content in wheat. (DOCX 18 kb) [file 12864_2016_3395_MOESM1_ESM.docx]

| **Table S1** List of detectd QTLs for yellow index and/or yellow pigment content in wheat. | | | | |
| --- | --- | --- | --- | --- |
| Chrom. | Marker, marker interval | Map position* | Variation explained (R^2^%) | References |
| 1AS | wmc469-cfd59 | 43.5-46.1 | 6-9 | Zhang *et al*., 2009b; *Zhao et al.,* 2013 |
| 1AL | cfa2147-gwm99 | 120-127.4 | 5-20 | Patil *et al*., 2008; Zhang *et al.*, 2008; Zhao *et al*., 2013 |
| 1BL | wmc626-barc302 | 34.6-53.7 | 5-11 | Roncallo *et al.*, 2012; Zhao et al., 2013; Zhai *et al*., 2015; |
| 1BL | IWB73028 | 100-110 | 13-27 | Colasuonno *et al*., 2014 |
| 1BL | IWB62262 | 140-152 | 6-12 | Zhao *et al*., 2013; Zhai *et al.*, 2015 |
| 2AS | gwm425-gwm372 | 99-107.7 | 11-24 | Pozniak *et al.*, 2007; Blanco *et al*., 2011; Colasuonno *et al*., 2014; Zhai *et al*., 2015 |
| 2AL | IWB58832 | 143-147 | 6-10 | Zhai *et al.*, 2015 |
| 2AL | IWB51274 | 196-207 | 7-8 | Zhai *et al.*, 2015 |
| 2BS | wmc382-wmc764 | 0-2.8 | 5 | Zhang *et al.*, 2009a |
| 2BS | IWB9689 | 70-75 | 10-13 | Zhai *et al.*, 2015 |
| 2BL | IWB73809 | 94-113 | 10-16 | Colasuonno *et al*., 2014; Zhai *et al*., 2015 |
| 3AS | barc57-IWB29389 | 5.6-9.6 | 11-13 | Parker et al., 1998; Colasuonno et al., 2014 |
| 3AS | gwm666 | 60-70 | 21 | Crawford et al., 2011a |
| 3BS | barc218 | 30-35 | 7 | Patil et al., 2008 |
| 3BS | gwm566-gwm285 | 59-76.9 | - | Mares and Campbell, 2001; Howitt *et al*., 2009 |
| 3BL | gwm299-barc84 | 192-198 | 9-17 | Blanco *et al*., 2011 |
| 4AL | gwm192-wmc617 | 63.6-81 | 12 | Roncallo *et al.*, 2012 |
| 4AL | gwm160-barc52 | 160-172 | 4-12 | Zhang *et al*., 2008; Zhang *et al*., 2009b; Roncallo et al., 2012; Zhao *et al*. 2013 |
| 4BS | wmc617-IWA7311 | 15-20 | 9-10 | Zhang and Dubcovsky, 2008; Zhai *et al*., 2015 |
| 4BS | gwm495 | 60-65 | - | Pozniak *et al*., 2007 |
| 5AS | IWA7361 | 12-17 | 7-9 | Zhai *et al.*, 2015 |
| 5AS | gwm304-IWB73092 | 45-60 | 7-18 | Zhang *et al*., 2009a; Blanco *et al*., 2011; Roncallo *et al.*, 2012; Colasuonno *et al.*, 2014 |
| 5AL | IWB26864 | 84-91.6 | 6-9 | Zhai *et al*., 2015 |
| 5BL | gwm540-barc74 | 38-56 | 6-14 | Howitt *et al*., 2009; Crawford *et al.*, 2011a; Tsilo *et al*., 2011; Zhao *et al.,*2013; Zhai *et al.*, 2015 |
| 5BL | gwm499-BE495277 | 90-94 | 9 | Roncallo *et al*., 2012 |
| 5BL | gwm408-barc232 | 140.3-146.3 | 8-22 | Patil *et al*., 2008; Tsilo *et al*., 2011; Colasuonno *et al.*, 2014 |
| 6AL | gwm132 | 53-63 | 23 | Roncallo *et al*., 2012 |
| 6AL | barc113-wmc553 | 73-95 | 7-43 | Zhang and Dubcovsky, 2008; Zhang *et al.*, 2008; Roncallo *et al*., 2012; Zhao *et al.,* 2013 |
| 6AL | BE483091_472 | 114-124 | 10 | Roncallo *et al*., 2012 |
| 6BL | gwm193-wmc539 | 74-90 | 7-9 | Pozniak *et al*., 2007; Zhai *et al*., 2015 |
| 6BL | IWB4109 | 131.8 | 8-11 | Zhai *et al*., 2015 |
| 7AS | IWB38528 | 1-5 | 6-14 | Zhai *et al*., 2015; Roncallo *et al*., 2012 |
| 7AL | barc174 | 90-110 | 7 | Elouafi *et al*., 2002 |
| 7AL | gwn282-IWB59875 | 170-183 | 13-60 | Zhang and Dubcovsky, 2008; Zhang *et al*., 2008; Blanco *et al.*, 2011; Crawford *et al*., 2011a; Colasuonno *et al.*, 2014 |
| 7AL | cfa2040-gwm344 | 192-206 | 13-60 | Parker *et al*., 1998; Mares and Campbell, 2001; Elouafi *et al.*, 2002; He *et al.*, 2008; Patil *et al.*, 2008; Singh *et al.,* 2009; He *et al*., 2009b; Howitt *et al.,* 2009; Zhang *et al*., 2009b; Blanco *et al.*, 2011; Crawford *et al.*, 2011a; Roncallo *et al*., 2012; Crawford and Francki, 2013b; Zhai *et al*., 2015; Campos *et al*., 2016 |
| 7BS | IWB34468 | 0-5 | 6-8 | Zhai *et al*., 2015 |
| 7BS | wmc546-wmc335 | 55-73 | 9-15 | Patil *et al*., 2008; Crawford *et al.*, 2011a; Roncallo *et al*., 2012 |
| 7BL | IWB6544 | 161-167 | 4-29 | Zhao *et al.* 2013; Colasuonno *et al*., 2014 |
| 7BL | wmc276-cfa2257 | 181-208 | 7-15 | Elouafi *et al.,* 2002; Pozniak *et al*., 2007; Zhang *et al.,* 2008; Howitt *et al*., 2009; He *et al*., 2009b; Crawford *et al.*, 2011a; Roncallo *et al*., 2012; Crawford and Francki, 2013b; Zhai *et al.*, 2015, |
| 2DS | wmc25-gwm484 |  | 16-17 | Zhang *et al.*, 2009b; Zhao *et al*. 2013 |
| 2DL | IWB51778 | 82.8 | 13-17 | Zhai *et al*., 2015 |
| 3DL | wmc631-barc323 |  | 2.9 | Zhang *et al.*, 2009a |
| 4DS | wmc89 -wmc457 |  | 4-6 | Crawford *et al.*, 2011a |
| 4DS | wPt2379-gwm194 |  | 16.5 | Zhao et al. 2013 |
| 4DS | rht2-wmc48 |  | 10 | Crawford *et al*., 2011a |
| 4DL | cfa2173-cfe188 |  | 3.4 | Zhang  *et al*., 2009a |
| 5DS | barc130-cfd18 |  | 13-16 | Tsilo *et al*., 2011 |
| 5DL | wPt5505-gwm182 |  | 14 | Zhao *et al.,* 2013 |
| 6D | barc21-wPt667726 |  | 4.1 | Zhao *et al*., 2013 |
| 7DS | IWB44453 | 102.1 | 9-10 | Zhai *et al.*, 2015 |
| *Map position of QTLs on chromosome 1A-7B is based on the SSR- and SNP-based consensus durum map published by Maccaferri et al. (2014). SSR markers in common between the consensus map and the specific QTL mapping study were used as anchor points. Map position of some QTLs on chromosome 1D-7D is based on the SNP-based consensus bread wheat map published by Wang et al. (2014). The position of the listed QTLs is illustrated on Fig. S3. | | | | |
