# Supplementary material for: The carotenoid biosynthetic and catabolic genes in wheat and their association with yellow pigments
Source: BMC Genomics. 2017 Jan 31;18:122. doi: 10.1186/s12864-016-3395-6 (PMC5286776; doi:10.1186/s12864-016-3395-6)
Supplement: Additional file 2: Figure S1. — Expression analysis from PLEXdb database of all key genes in carotenoid biosynthesis. (DOCX 83 kb) [file 12864_2016_3395_MOESM2_ESM.docx]

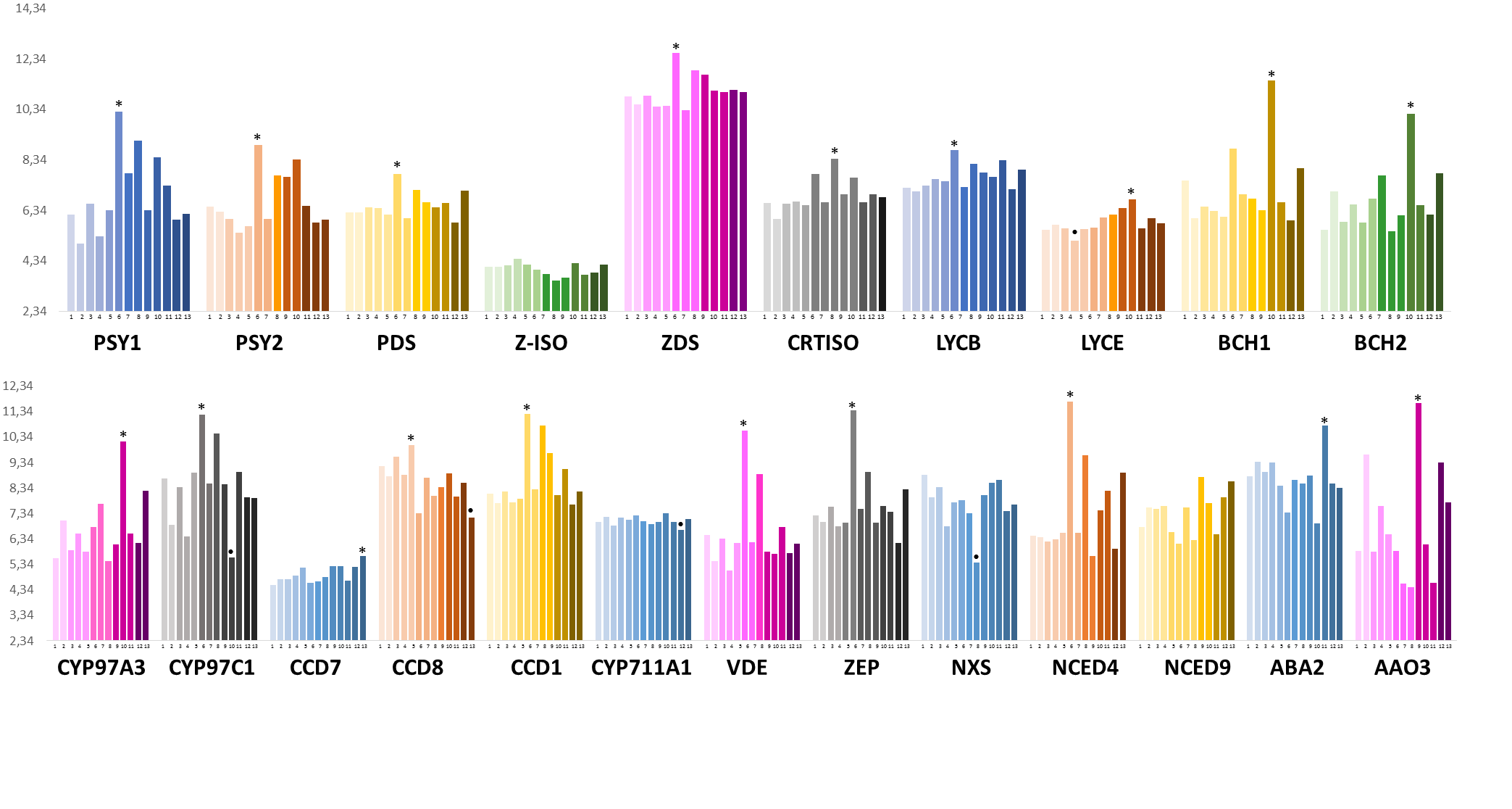


**Developmental stages**:

**1** germinating seed, coleoptile **4** seedling, root **7** immature inflorescence **10** anthers, before anthesis

**2** germinating seed, root **5** seedling, crown **8** floral bracts, before anthesis **11** 3-5 DAP caryopsis

**3** germinating seed, embryo **6** seedling, leaf **9** pistil, before anthesis **12** 22 DAP embryo

**13** 22 DAP endosperm

**Figure S1.** Expression analysis from PLEXdb database of all key genes in carotenoid biosynthesis. The data of bread wheat cv. *Chinese Spring* are referred to a wide range of tissues and developmental stages (from 1 to 13) (x axes) in RMA normalization procedure (y axes) obtained by the Affymetrix Wheat Gene Chip. The asterisk and dot signals indicate respectively the values higher or lower than the mean values ± 2 SD.
