# Supplementary material for: The carotenoid biosynthetic and catabolic genes in wheat and their association with yellow pigments
Source: BMC Genomics. 2017 Jan 31;18:122. doi: 10.1186/s12864-016-3395-6 (PMC5286776; doi:10.1186/s12864-016-3395-6)
Supplement: Additional file 3: Figure S2. — Frequency distributions of yellow index and yellow pigment content. (DOCX 28 kb) [file 12864_2016_3395_MOESM3_ESM.docx]

a)

d)

c)

b)

Fig. S2 Frequency distributions of yellow pigment content in the whole wheat collection (a) and in the durum sub-population (b), and of yellow index in the whole collection (c) and in the durum sub-population (d).
