# Supplementary material for: The carotenoid biosynthetic and catabolic genes in wheat and their association with yellow pigments
Source: BMC Genomics. 2017 Jan 31;18:122. doi: 10.1186/s12864-016-3395-6 (PMC5286776; doi:10.1186/s12864-016-3395-6)
Supplement: Additional file 4: Table S2. — Correlation coefficients of yellow pigment content and of yellow index. (DOCX 12 kb) [file 12864_2016_3395_MOESM4_ESM.docx]

| **Table S2.** Correlation coefficients of yellow pigment content evaluated in two environments and of yellow index evaluated in six environments in the whole collection. | | | | | |
| --- | --- | --- | --- | --- | --- |
|  | Foggia 2009 | Valenzano 2010 | Foggia 2012 | Valenzano 2012 | Valenzano 2013 |
| *Yellow Pigment Content* |  |  |  |  |  |
| Foggia 2008 | 0.92*** |  |  |  |  |
|  |  |  |  |  |  |
| *Yellow Index* |  |  |  |  |  |
| Valenzano 2010 | 0.84*** |  |  |  |  |
| Foggia 2012 | 0.77*** | 0.81*** |  |  |  |
| Valenzano 2012 | 0.86*** | 0.90*** | 0.78*** |  |  |
| Valenzano 2013 | 0.83*** | 0.92*** | 0.82*** | 0.87*** |  |
| Valenzano 2014 | 0.81*** | 0.91*** | 0.77*** | 0.85*** | 0.89*** |
| ***: significant differences at 0.001P | |  |  |  |  |
