# Supplementary material for: The carotenoid biosynthetic and catabolic genes in wheat and their association with yellow pigments
Source: BMC Genomics. 2017 Jan 31;18:122. doi: 10.1186/s12864-016-3395-6 (PMC5286776; doi:10.1186/s12864-016-3395-6)
Supplement: Additional file 5: Table S3. — Number of significant marker-trait associations detected by four GWAS models. (DOCX 13 kb) [file 12864_2016_3395_MOESM5_ESM.docx]

| **Table S3.** Number of marker-trait associations significant at -log_10_(P)≥3 detected by four GWAS models in the whole collection and in the durum sub-population evaluated for yellow index (mean of six environments) and yellow pigment content (mean of two environments). | | | | | | | | | | | |
| --- | --- | --- | --- | --- | --- | --- | --- | --- | --- | --- | --- |
|  | Whole wheat collection | | | | |  | Durum sub-population | | | | |
|  | Yellow index | |  | Yellow pigment content | | | Yellow index | |  | Yellow pigment content | |
|  | n. | (%) |  | n. | (%) |  | n. | (%) |  | n. | (%) |
| GLM | 3286 | (24.1) |  | 5548 | (40.7) |  | 718 | (7.3) |  | 689 | (7.0) |
| GLM+PCs | 1175 | (8.6) |  | 1267 | (9.3) |  | 143 | (1.4) |  | 89 | (0.9) |
| MLM+K | 34 | (0.2) |  | 19 | (0.1) |  | 35 | (0.4) |  | 25 | (0.3) |
| MLM+K+PCs | 28 | (0.2) |  | 17 | (0.1) |  | 36 | (0.4) |  | 27 | (0.3) |
