# Supplementary material for: The carotenoid biosynthetic and catabolic genes in wheat and their association with yellow pigments
Source: BMC Genomics. 2017 Jan 31;18:122. doi: 10.1186/s12864-016-3395-6 (PMC5286776; doi:10.1186/s12864-016-3395-6)
Supplement: Additional file 6: Figure S3. — Genome-wide association analysis for yellow index and yellow pigment content: Q-Q plots. (DOCX 1809 kb) [file 12864_2016_3395_MOESM6_ESM.docx]

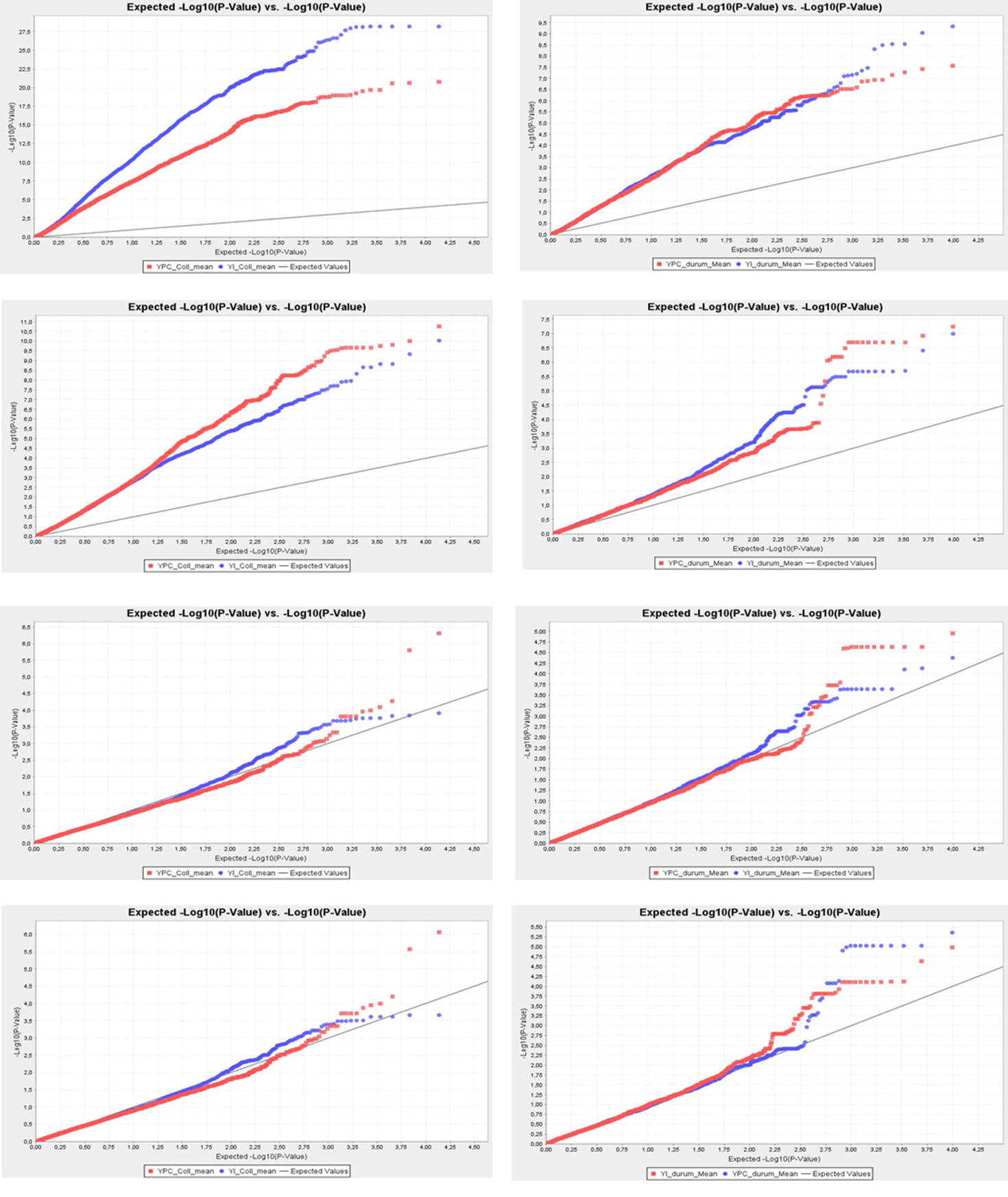


**h)**

**g)**

**f)**

**e)**

**d)**

**c)**

**a)**

**b)**

**Whole wheat collection**

**Durum sub-population**

**Figure S3.** Genome-wide association analysis for yellow index and yellow pigment content in the whole wheat collection and in the durum sub-population (mean across environments). Quantile-quantile (Q-Q) plots of the observed –log10 (P) values (y axes) against the expected distribution of –log10 (P) values (x axes) for: GLM (a, b), GLM (c, d), MLM+K (e, f), MLM+K+PCs (g, h) models.
